# *trans*-Di­chlorido­tetra­kis­(4-meth­oxy­pyridine-κ*N*)ruthenium(II)

**DOI:** 10.1107/S2414314623001554

**Published:** 2023-02-28

**Authors:** Eric W. Reinheimer, Rebecca A. Tobias, Elisabeth R. Bassel, Nicholas A. Cantu, Bradley W. Smucker

**Affiliations:** a Rigaku Americas Corporation, 9009 New Trails Dr., The Woodlands, TX 77381, USA; b 900 N Grand Avenue, Suite 61651, Sherman, TX 75090, USA; Howard University, USA

**Keywords:** crystal structure, ruthenium(II), 4-meth­oxy­pyridine

## Abstract

The structure of the title complex, [RuCl_2_(C_6_H_6_NO)_4_], exhibits point group symmetry  . The structure exhibits disorder around a  axis. The 4-meth­oxy­pyridine ligands have a propeller-like arrangement around the Ru^II^ atom at 52.0 (3)° from the RuN_4_ plane.

## Abstract 2

The structure of the title compound exhibits disorder around a 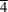 axis. The 4-meth­oxy­pyridine ligands have a propeller-like arrangement around the ruthenium at 52.01° from the RuN_4_ plane.

## Graphical abstract

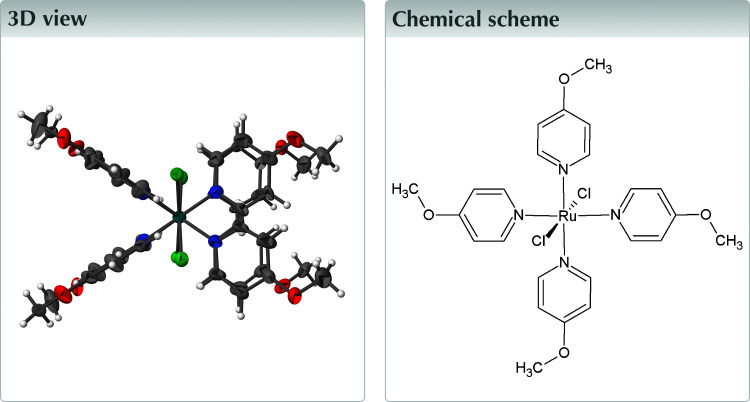

## Structure description

**Figure 1:**
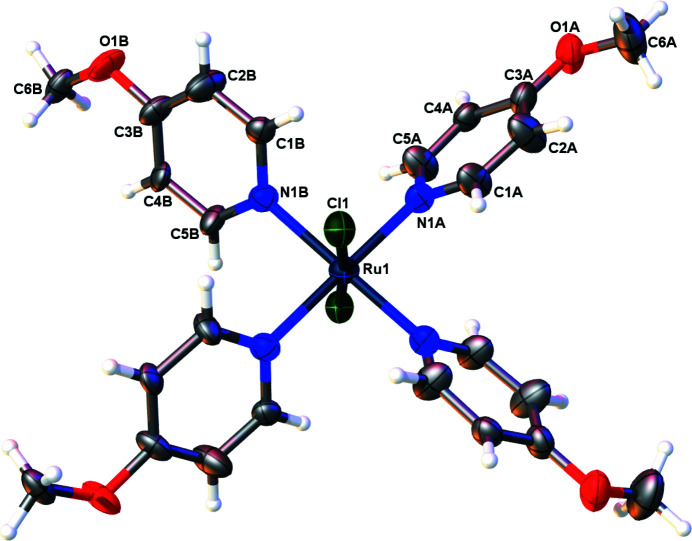
Displacement ellipsoid (50% probability level) representation of the title complex with disorder omitted for clarity.

**Figure 2:**
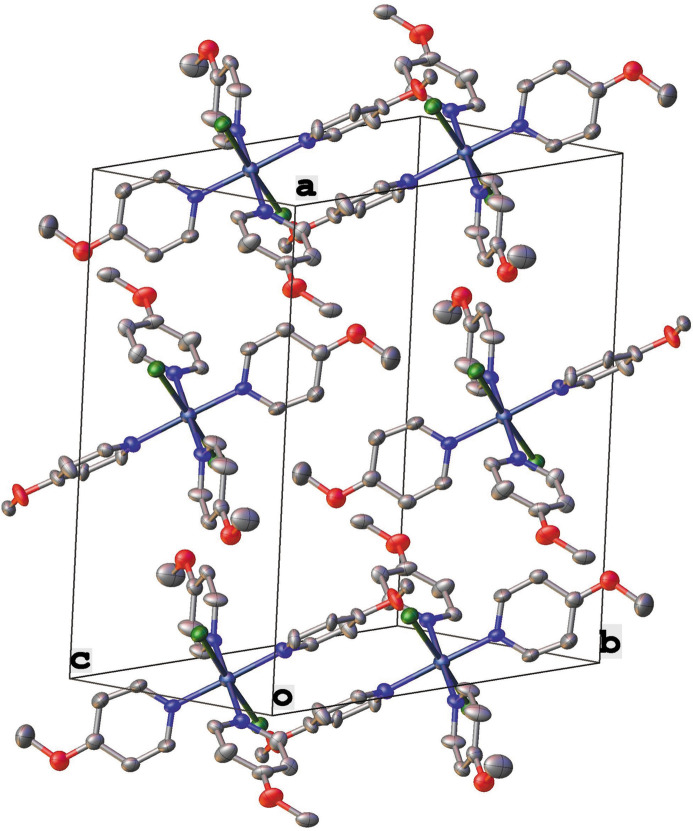
Projection of **1** onto the (111) plane. Anisotropic displacement ellipsoids have been set to the 50% probability level. Additional conformations of the mol­ecule generated *via* the disorder around 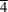 as well as the hydrogen atoms have been removed for the sake of clarity.

The Ru—N distances in the title compound (Fig. 1 ▸) are 2.059 (7) and 2.137 (5) Å for N1*A* and N1*B*, respectively. These diverge from the Ru—N(pyrid­yl) distances of 2.090 (3) and 2.092 (3) Å found in the structure of the ruthenium(II) complex containing four 4-meth­oxy­pyridine and *trans*-bis­(thio­cyanato-κ*N*) ligands (Cadranel *et al.*, 2016 ▸). The title complex has a propeller-like arrangement of the pyridyl ligands around the ruth­enium(II) at 52.0 (3)° from the plane containing the ruthenium and the coordinating nitro­gen atoms. This arrangement is typical of Ru^II^ complexes with polypyridyl ligands such as the aforementioned bis­(thio­cyanato) complex (Cadranel *et al.*, 2016 ▸) or Ru(pyrazine-κ*N*)_4_Cl_2_ (Nesterov *et al.*, 2012 ▸). The structure of the title complex has the ruthenium atoms positioned on the 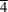 axis (Fig. 2 ▸), which results in disorder of the chlorido and 4-methoxypyridine ligands.

## Synthesis and crystallization

Following the synthetic procedures for *trans*-Ru(4-meth­oxy­pyridine-κ*N*)_4_Cl_2_ (Alborés *et al.*, 2004 ▸) and *trans*-Ru(pyrazine-κ*N*)_4_Cl_2_ (Carlucci *et al.*, 2002 ▸), a mixture of 4-meth­oxy­pyridine (0.5 mL, 5 mmol) and [RuCl_2_(dmso)_4_] (100 mg, 0.21 mmol) in 17 mL of toluene and 3 mL of butanol were refluxed for 3 h with stirring. After sitting in the cooled solution for four days, the solid was filtered in air and washed with 20 mL of toluene to afford 49 mg of the product (39% yield).

Orange prisms were grown from a slow liquid diffusion of tetra­hydro­furan into a di­chloro­methane solution of the title complex.

## Refinement

Crystal data, data collection, and refinement details are summarized in Table 1 ▸. The asymmetric unit contains one 4-meth­oxy­pyridine disordered over two positions around the 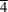 axis with ratios set to 0.55 and 0.45 between the two conformations. This ratio yielded the highest quality model as judged by the metrics *R*1, *wR*2, as well as resolution of residual electron density. Standard uncertainties were not reported due to the occupancy ratios being fixed. H atoms bound to C atoms were positioned geometrically (C—H = 0.93 or 0.96 Å) and constrained to ride on the parent atom. *U*_iso_ (H) values were set to a multiple of *U*_eq_ (C) [1.2 for CH_2_ (*sp^2^*) and 1.5 for CH_3_ (*sp^3^*)]. Twinning by merohedry was resolved by completing the final refinement using the matrix (0 1 0 1 0 0 0 0 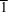 ) twin law.

## Supplementary Material

Crystal structure: contains datablock(s) I. DOI: 10.1107/S2414314623001554/bv4046sup1.cif

Structure factors: contains datablock(s) I. DOI: 10.1107/S2414314623001554/bv4046Isup2.hkl

CCDC reference: 2243464

Additional supporting information:  crystallographic information; 3D view; checkCIF report

## Figures and Tables

**Table 1:** Experimental details

Crystal data	
Chemical formula	[RuCl_2_(C_12_H_14_N_2_O_2_)_2_]
*M*_r_	608.47
Crystal system, space group	Tetragonal, *I*4_1_/*a*
Temperature (K)	293
*a*, *c* (Å)	17.2417 (1), 8.7307 (2)
*V* (Å^3^)	2595.43 (7)
*Z*	4
Radiation type	Mo *K*α
μ (mm^−1^)	0.85
Crystal size (mm)	0.25 × 0.12 × 0.10

Data collection	
Diffractometer	XtaLAB Mini II
Absorption correction	Multi-scan (*CrysAlis PRO*; Rigaku OD, 2022 ▸)
*T*_min_, *T*_max_	0.901, 1.000
No. of measured, independent and observed [*I* > 2σ(*I*)] reflections	102632, 1494, 1367
*R*_int_	0.029
(sin θ/λ)_max_ (Å^−1^)	0.649

Refinement	
*R*[*F* ^2^ > 2σ(*F* ^2^)], *wR*(*F* ^2^), *S*	0.030, 0.082, 1.07
No. of reflections	1494
No. of parameters	147
No. of restraints	131
H-atom treatment	H-atom parameters constrained
Δρ_max_, Δρ_min_ (e Å^−3^)	0.38, −0.38

Computer programs: *CrysAlis PRO* (Rigaku OD, 2022 ▸), *SHELXT* (Sheldrick, 2015*a* ▸), *SHELXL2016/6* (Sheldrick, 2015*b* ▸), and *OLEX2* (Dolomanov *et al.*, 2009 ▸).

## full crystallographic data

### Crystal data

**Table:**

[RuCl_2_(C_12_H_14_N_2_O_2_)_2_]	*D*_x_ = 1.557 Mg m^−^^3^
*M**_r_* = 608.47	Mo *K*α radiation, λ = 0.71073 Å
Tetragonal, *I*4_1_/*a*	Cell parameters from 17604 reflections
*a* = 17.2417 (1) Å	θ = 2.4–30.1°
*c* = 8.7307 (2) Å	µ = 0.85 mm^−^^1^
*V* = 2595.43 (7) Å^3^	*T* = 293 K
*Z* = 4	Block, orange
*F*(000) = 1240	0.25 × 0.12 × 0.1 mm

### Data collection

**Table:**

XtaLAB Mini II diffractometer	1494 independent reflections
Radiation source: fine-focus sealed X-ray tube, Rigaku (Mo) X-ray Source	1367 reflections with *I* > 2σ(*I*)
Graphite monochromator	*R*_int_ = 0.029
Detector resolution: 10.0000 pixels mm^-1^	θ_max_ = 27.5°, θ_min_ = 2.6°
ω scans	*h* = −22→22
Absorption correction: multi-scan (CrysAlisPro; Rigaku OD, 2022)	*k* = −22→22
*T*_min_ = 0.901, *T*_max_ = 1.000	*l* = −11→11
102632 measured reflections	

### Refinement

**Table:**

Refinement on *F*^2^	Primary atom site location: dual
Least-squares matrix: full	Hydrogen site location: inferred from neighbouring sites
*R*[*F*^2^ > 2σ(*F*^2^)] = 0.030	H-atom parameters constrained
*wR*(*F*^2^) = 0.082	*w* = 1/[σ^2^(*F*_o_^2^) + 15.430*P*] where *P* = (*F*_o_^2^ + 2*F*_c_^2^)/3
*S* = 1.07	(Δ/σ)_max_ < 0.001
1494 reflections	Δρ_max_ = 0.38 e Å^−^^3^
147 parameters	Δρ_min_ = −0.38 e Å^−^^3^
131 restraints	

### Special details

**Table:**

Geometry. All esds (except the esd in the dihedral angle between two l.s. planes) are estimated using the full covariance matrix. The cell esds are taken into account individually in the estimation of esds in distances, angles and torsion angles; correlations between esds in cell parameters are only used when they are defined by crystal symmetry. An approximate (isotropic) treatment of cell esds is used for estimating esds involving l.s. planes.
Refinement. Refined as a 2-component twin.

### Fractional atomic coordinates and isotropic or equivalent isotropic displacement parameters (Å^2^)

**Table:**

	*x*	*y*	*z*	*U*_iso_*/*U*_eq_	Occ. (<1)
Ru1	0.500000	0.250000	0.875000	0.03156 (14)	
N1B	0.4401 (5)	0.3133 (5)	0.7009 (8)	0.0333 (16)	0.45
C1B	0.3596 (5)	0.3168 (5)	0.6994 (9)	0.031 (2)	0.45
H1B	0.331203	0.293777	0.777710	0.038*	0.45
C2B	0.3214 (4)	0.3547 (7)	0.5807 (11)	0.053 (4)	0.45
H2B	0.267559	0.357028	0.579691	0.064*	0.45
C3B	0.3639 (6)	0.3891 (6)	0.4636 (9)	0.038 (2)	0.45
C4B	0.4444 (6)	0.3857 (5)	0.4652 (8)	0.033 (2)	0.45
H4B	0.472767	0.408712	0.386851	0.040*	0.45
C5B	0.4825 (4)	0.3478 (6)	0.5838 (10)	0.036 (3)	0.45
H5B	0.536412	0.345461	0.584869	0.043*	0.45
Cl1	0.3999 (6)	0.3486 (6)	0.8850 (3)	0.0360 (4)	0.5
O1B	0.3229 (10)	0.4262 (12)	0.3520 (15)	0.052 (4)	0.45
N1A	0.4418 (6)	0.3089 (5)	1.0449 (8)	0.0321 (13)	0.55
C5A	0.4350 (7)	0.3866 (6)	1.0540 (13)	0.045 (3)	0.55
H5A	0.459380	0.415337	0.977769	0.054*	0.55
C2A	0.3646 (7)	0.3066 (7)	1.2771 (14)	0.055 (4)	0.55
H2A	0.341706	0.275510	1.351489	0.066*	0.55
C1A	0.4062 (7)	0.2740 (6)	1.1595 (13)	0.044 (3)	0.55
H1A	0.409791	0.220231	1.160770	0.052*	0.55
C3A	0.3574 (7)	0.3869 (7)	1.2831 (8)	0.038 (2)	0.55
C4A	0.3959 (6)	0.4277 (5)	1.1639 (11)	0.035 (2)	0.55
H4A	0.394618	0.481609	1.160556	0.042*	0.55
C6A	0.2873 (11)	0.3862 (10)	1.5143 (15)	0.077 (5)	0.55
H6AA	0.247065	0.353501	1.474172	0.116*	0.55
H6AB	0.265534	0.421274	1.588180	0.116*	0.55
H6AC	0.326225	0.354836	1.562464	0.116*	0.55
O1A	0.3210 (10)	0.4290 (9)	1.3939 (10)	0.051 (3)	0.55
C6B	0.3627 (9)	0.4650 (8)	0.2338 (13)	0.041 (3)	0.45
H6BA	0.326401	0.493663	0.172646	0.061*	0.45
H6BB	0.389015	0.427743	0.170615	0.061*	0.45
H6BC	0.399952	0.499992	0.277467	0.061*	0.45

### Atomic displacement parameters (Å^2^)

**Table:**

	*U*^11^	*U*^22^	*U*^33^	*U*^12^	*U*^13^	*U*^23^
Ru1	0.02965 (17)	0.02965 (17)	0.0354 (3)	0.000	0.000	0.000
N1B	0.034 (3)	0.030 (5)	0.036 (3)	0.004 (3)	0.000 (3)	0.004 (3)
C1B	0.034 (3)	0.026 (6)	0.033 (4)	0.006 (3)	−0.001 (3)	−0.002 (4)
C2B	0.040 (4)	0.056 (10)	0.063 (6)	0.003 (5)	−0.008 (4)	0.027 (7)
C3B	0.038 (4)	0.030 (6)	0.047 (5)	0.003 (4)	−0.014 (3)	0.011 (5)
C4B	0.039 (4)	0.041 (6)	0.020 (4)	0.002 (4)	−0.012 (3)	−0.005 (3)
C5B	0.032 (4)	0.048 (7)	0.029 (4)	−0.004 (4)	−0.010 (3)	0.007 (4)
Cl1	0.032 (2)	0.042 (3)	0.0339 (8)	0.0063 (6)	−0.0015 (13)	0.0008 (13)
O1B	0.047 (6)	0.055 (9)	0.054 (6)	0.009 (5)	−0.018 (5)	0.022 (6)
N1A	0.039 (4)	0.023 (3)	0.034 (2)	0.001 (2)	0.001 (2)	0.007 (2)
C5A	0.058 (7)	0.023 (3)	0.053 (5)	−0.001 (3)	0.018 (5)	0.006 (3)
C2A	0.068 (8)	0.035 (3)	0.061 (6)	0.004 (4)	0.029 (6)	0.013 (3)
C1A	0.056 (7)	0.025 (3)	0.049 (4)	0.004 (4)	0.016 (4)	0.013 (3)
C3A	0.050 (6)	0.036 (3)	0.030 (3)	0.003 (3)	−0.005 (3)	0.005 (3)
C4A	0.041 (6)	0.028 (3)	0.034 (3)	0.005 (3)	−0.005 (3)	0.006 (3)
C6A	0.117 (13)	0.070 (9)	0.046 (6)	0.008 (8)	0.029 (7)	0.006 (6)
O1A	0.073 (9)	0.049 (5)	0.032 (4)	0.006 (4)	0.003 (5)	−0.001 (4)
C6B	0.056 (7)	0.029 (5)	0.037 (5)	0.005 (5)	−0.020 (5)	0.003 (4)

### Geometric parameters (Å, º)

**Table:**

Ru1—N1B^i^	2.137 (5)	C4B—C5B	1.3900
Ru1—N1B^ii^	2.137 (5)	C5B—H5B	0.9300
Ru1—N1B^iii^	2.137 (5)	O1B—C6B	1.409 (11)
Ru1—N1B	2.137 (5)	N1A—C5A	1.346 (9)
Ru1—Cl1^iii^	2.4235 (15)	N1A—C1A	1.319 (10)
Ru1—Cl1^ii^	2.4235 (15)	C5A—H5A	0.9300
Ru1—Cl1^i^	2.4235 (16)	C5A—C4A	1.370 (11)
Ru1—Cl1	2.4236 (15)	C2A—H2A	0.9300
Ru1—N1A	2.059 (7)	C2A—C1A	1.372 (12)
Ru1—N1A^iii^	2.059 (7)	C2A—C3A	1.391 (11)
Ru1—N1A^i^	2.059 (7)	C1A—H1A	0.9300
Ru1—N1A^ii^	2.059 (7)	C3A—C4A	1.421 (11)
N1B—C1B	1.3900	C3A—O1A	1.363 (7)
N1B—C5B	1.3900	C4A—H4A	0.9300
C1B—H1B	0.9300	C6A—H6AA	0.9600
C1B—C2B	1.3900	C6A—H6AB	0.9600
C2B—H2B	0.9300	C6A—H6AC	0.9600
C2B—C3B	1.3900	C6A—O1A	1.409 (11)
C3B—C4B	1.3900	C6B—H6BA	0.9600
C3B—O1B	1.363 (7)	C6B—H6BB	0.9600
C4B—H4B	0.9300	C6B—H6BC	0.9600
			
N1B^i^—Ru1—N1B^ii^	120.4 (3)	N1A^ii^—Ru1—N1A^i^	121.2 (2)
N1B^i^—Ru1—N1B	120.4 (3)	N1A—Ru1—N1A^iii^	121.2 (2)
N1B^ii^—Ru1—N1B	89.4 (5)	N1A^iii^—Ru1—N1A^i^	87.9 (4)
N1B^iii^—Ru1—N1B	120.4 (3)	C1B—N1B—Ru1	120.8 (5)
N1B^iii^—Ru1—Cl1^ii^	90.0 (4)	C1B—N1B—C5B	120.0
N1B^iii^—Ru1—Cl1^i^	136.7 (2)	C5B—N1B—Ru1	119.1 (5)
N1B^i^—Ru1—Cl1^ii^	87.1 (4)	N1B—C1B—H1B	120.0
N1B^ii^—Ru1—Cl1^i^	90.0 (4)	N1B—C1B—C2B	120.0
N1B^i^—Ru1—Cl1^i^	47.4 (2)	C2B—C1B—H1B	120.0
N1B^ii^—Ru1—Cl1^ii^	47.4 (2)	C1B—C2B—H2B	120.0
N1B^iii^—Ru1—Cl1	87.1 (4)	C1B—C2B—C3B	120.0
N1B^ii^—Ru1—Cl1	136.7 (2)	C3B—C2B—H2B	120.0
N1B—Ru1—Cl1^i^	87.1 (4)	C4B—C3B—C2B	120.0
N1B—Ru1—Cl1	47.4 (2)	O1B—C3B—C2B	117.0 (9)
N1B—Ru1—Cl1^ii^	136.7 (2)	O1B—C3B—C4B	123.0 (9)
N1B^i^—Ru1—Cl1	90.0 (4)	C3B—C4B—H4B	120.0
Cl1^i^—Ru1—Cl1^ii^	90.075 (5)	C3B—C4B—C5B	120.0
Cl1^i^—Ru1—Cl1	90.076 (5)	C5B—C4B—H4B	120.0
Cl1^ii^—Ru1—Cl1	175.86 (12)	N1B—C5B—H5B	120.0
Cl1^iii^—Ru1—Cl1^ii^	90.075 (5)	C4B—C5B—N1B	120.0
Cl1^iii^—Ru1—Cl1	90.074 (5)	C4B—C5B—H5B	120.0
Cl1^iii^—Ru1—Cl1^i^	175.86 (12)	C3B—O1B—C6B	119.6 (13)
N1A—Ru1—N1B^i^	59.9 (2)	C5A—N1A—Ru1	125.2 (7)
N1A^i^—Ru1—N1B^iii^	178.8 (3)	C1A—N1A—Ru1	123.2 (6)
N1A^ii^—Ru1—N1B^iii^	59.9 (2)	C1A—N1A—C5A	111.6 (7)
N1A^iii^—Ru1—N1B^ii^	58.5 (2)	N1A—C5A—H5A	116.6
N1A^ii^—Ru1—N1B^i^	58.5 (2)	N1A—C5A—C4A	126.8 (8)
N1A—Ru1—N1B^ii^	178.8 (3)	C4A—C5A—H5A	116.6
N1A^ii^—Ru1—N1B^ii^	91.40 (18)	C1A—C2A—H2A	120.6
N1A^iii^—Ru1—N1B^i^	178.8 (3)	C1A—C2A—C3A	118.8 (8)
N1A^i^—Ru1—N1B^i^	91.40 (18)	C3A—C2A—H2A	120.6
N1A^i^—Ru1—N1B^ii^	59.9 (2)	N1A—C1A—C2A	128.6 (8)
N1A—Ru1—N1B^iii^	58.5 (2)	N1A—C1A—H1A	115.7
N1A^iii^—Ru1—N1B^iii^	91.40 (18)	C2A—C1A—H1A	115.7
N1A^ii^—Ru1—Cl1^iii^	90.9 (4)	C2A—C3A—C4A	115.1 (8)
N1A—Ru1—Cl1^ii^	131.8 (2)	O1A—C3A—C2A	126.7 (11)
N1A^iii^—Ru1—Cl1^ii^	92.0 (4)	O1A—C3A—C4A	118.1 (10)
N1A^i^—Ru1—Cl1	92.0 (4)	C5A—C4A—C3A	119.1 (7)
N1A—Ru1—Cl1	44.0 (2)	C5A—C4A—H4A	120.4
N1A^i^—Ru1—Cl1^ii^	90.9 (4)	C3A—C4A—H4A	120.4
N1A^ii^—Ru1—Cl1	131.9 (2)	H6AA—C6A—H6AB	109.5
N1A^i^—Ru1—Cl1^iii^	131.8 (2)	H6AA—C6A—H6AC	109.5
N1A^iii^—Ru1—Cl1^iii^	44.0 (2)	H6AB—C6A—H6AC	109.5
N1A^ii^—Ru1—Cl1^i^	92.0 (4)	O1A—C6A—H6AA	109.5
N1A^iii^—Ru1—Cl1	90.9 (4)	O1A—C6A—H6AB	109.5
N1A—Ru1—Cl1^i^	90.9 (4)	O1A—C6A—H6AC	109.5
N1A^ii^—Ru1—Cl1^ii^	44.0 (2)	C3A—O1A—C6A	116.2 (12)
N1A^iii^—Ru1—Cl1^i^	131.8 (2)	O1B—C6B—H6BA	109.5
N1A—Ru1—Cl1^iii^	92.0 (4)	O1B—C6B—H6BB	109.5
N1A^i^—Ru1—Cl1^i^	44.0 (2)	O1B—C6B—H6BC	109.5
N1A—Ru1—N1A^ii^	87.9 (4)	H6BA—C6B—H6BB	109.5
N1A^iii^—Ru1—N1A^ii^	121.2 (2)	H6BA—C6B—H6BC	109.5
N1A—Ru1—N1A^i^	121.2 (2)	H6BB—C6B—H6BC	109.5
			
Ru1—N1B—C1B—C2B	−176.5 (7)	C5B—N1B—C1B—C2B	0.0
Ru1—N1B—C5B—C4B	176.6 (7)	O1B—C3B—C4B—C5B	179.1 (15)
Ru1—N1A—C5A—C4A	179.0 (9)	N1A—C5A—C4A—C3A	−0.1 (16)
Ru1—N1A—C1A—C2A	−178.7 (10)	C5A—N1A—C1A—C2A	1.3 (14)
N1B—C1B—C2B—C3B	0.0	C2A—C3A—C4A—C5A	1.0 (14)
C1B—N1B—C5B—C4B	0.0	C2A—C3A—O1A—C6A	0 (2)
C1B—C2B—C3B—C4B	0.0	C1A—N1A—C5A—C4A	−1.0 (14)
C1B—C2B—C3B—O1B	−179.2 (14)	C1A—C2A—C3A—C4A	−0.8 (14)
C2B—C3B—C4B—C5B	0.0	C1A—C2A—C3A—O1A	−177.4 (14)
C2B—C3B—O1B—C6B	177.3 (13)	C3A—C2A—C1A—N1A	−0.5 (16)
C3B—C4B—C5B—N1B	0.0	C4A—C3A—O1A—C6A	−176.6 (14)
C4B—C3B—O1B—C6B	−2 (3)	O1A—C3A—C4A—C5A	178.0 (12)

Symmetry codes: (i) *y*+1/4, −*x*+3/4, −*z*+7/4; (ii) −*x*+1, −*y*+1/2, *z*; (iii) −*y*+3/4, *x*−1/4, −*z*+7/4.
